# Ultra-Processed Foods Elicit Higher Approach Motivation Than Unprocessed and Minimally Processed Foods

**DOI:** 10.3389/fpubh.2022.891546

**Published:** 2022-06-21

**Authors:** Thayane C. Lemos, Guilherme M. S. Coutinho, Laiz A. A. Silva, Jasmin B. Stariolo, Rafaela R. Campagnoli, Leticia Oliveira, Mirtes G. Pereira, Bruna E. F. Mota, Gabriela G. L. Souza, Daniela S. Canella, Neha Khandpur, Isabel A. David

**Affiliations:** ^1^Department of Physiology and Pharmacology, Biomedical Institute, Universidade Federal Fluminense, Niterói, Brazil; ^2^Department of Neurobiology, Institute of Biology, Universidade Federal Fluminense, Niterói, Brazil; ^3^Laboratory of Psychophysiology, Department of Biological Sciences, Universidade Federal de Ouro Preto, Ouro Preto, Brazil; ^4^School of Nutrition, Universidade Federal de Ouro Preto, Ouro Preto, Brazil; ^5^Department of Applied Nutrition, Nutrition Institute, Universidade Do Estado Do Rio de Janeiro, Rio de Janeiro, Brazil; ^6^Department of Nutrition, Center for Epidemiological Research in Nutrition and Health, Universidade de São Paulo, São Paulo, Brazil; ^7^Department of Nutrition, Harvard T.H. Chan School of Public Health, Harvard University, Boston, MA, United States

**Keywords:** obesity, emotion, ultra-processed foods (UPFs), policy, marketing, food system

## Abstract

**Background:**

Ultra-processed foods (UPF) are becoming extensively available in the food environments. UPF are industrial formulations that are designed to maximize palatability and consumption through a combination of calorie-dense ingredients and chemical additives. UPFs are also aggressively marketed, which may make them more attractive than unprocessed/minimally processed foods (UMPF). Since consumers' purchase decisions are guided by food-evoked emotions, we aimed to provide evidence that UPF visual cues trigger higher emotional responses and approach motivation than UMPF visual cues, with potential impacts on individuals' intention to consume the UPF over the UMPF.

**Methods:**

Participants (*n* = 174; 144 women; mean age = 20.7 years; standard deviation = 4.35) performed two tasks. In the first task, 16 pictures of foods (8 UPF and 8 UMPF), and 74 pictures from other affective categories, were presented. After viewing each picture, the participants rated it along two basic dimensions of emotion through the Self-Assessment Manikin scale: pleasantness and arousal. In the second task, the participants viewed the same food pictures, and they rated their intention to consume the foods depicted in the pictures. Each picture was plotted in terms of its mean pleasantness and arousal ratings in a Cartesian plane, which resulted in an affective space.

**Results:**

Pictures of UPF and UMPF were positioned in the upper arm of the boomerang-shaped affective space that represents approach motivation. Pictures containing UPF triggered higher approach motivation and intention to consume than pictures containing UMPF. We also found a stronger association between emotional responses and intention to consume UPF relative to UMPF.

**Conclusion:**

These results shed new light on the role of ultra-processed foods evoked emotions that contribute to less healthy and sustainable food environments.

## Introduction

Obesity has become a global pandemic, reaching alarming levels over the entire planet (1). Recently, the obesity pandemic has been included as part of a Global Syndemic along with undernutrition and climate change, and the synergistic interactions between these conditions may increase the risk of adverse health outcomes (2). These three pandemics co-occur in time and place and share common underlying drivers, with a major driver being current food systems. The production, distribution and commercialization of ultra-processed foods (UPF) is an important driving force in the Global Syndemic since UPF contribute to food systems that are less healthy and less sustainable (3–5).

Ultra-processed foods, as defined by the NOVA food system classification, are industrial food and drink formulations that comprise several ingredients, including additives and food industry ingredients not used in home-based culinary preparations (6, 7). UPF have substantial negative impacts on planetary health by triggering biodiversity loss, the use of extensive packaging and waste production, and impacts on greenhouse gas emissions (3, 5) and are extensively available in the urban food environment (8, 9). The consumption of UPF has also been associated with adverse health outcomes such as the development of obesity and diet-related non-communicable diseases (4).

The response to the Global Syndemic requires the rethinking of all activities related to the production, processing, distribution, and consumption of food, that is, the rethinking of food systems (2). Consumer behavior is a point of leverage for food systems because of its impacts on market demands, which often determine what foods will be produced (10). Consumers' decisions toward foods are in turn influenced by food environments, which include the physical spaces where consumers interact with foods (11).

It has been demonstrated that food cues in the environment affect consumer behavior through the emotions that they evoke (12, 13). Thus, emotions can help us to understand consumers' food experiences and choices (14, 15). For instance, it has been shown that food-evoked emotions predict food choices better than food-liking alone (12, 16, 17). Indeed, the food industry has long associated their food products with positive emotional content to attract consumers (18–20). Food-evoked emotions may occur *via* consumers' interaction with extrinsic (e.g., package, brand, and marketing) and intrinsic (e.g., sensory food aspects) food attributes (12, 16, 21, 22). UPF may present very appealing intrinsic and extrinsic attributes because they are hyperpalatable and aggressively marketed (8).

From a psychological perspective, emotions may configure action dispositions toward positive, appetitive stimuli and away from negative, unpleasant stimuli (23, 24). This means that emotional cues activate motivational systems in the brain, namely, the appetitive and defensive systems, that promote individuals' survival through approach and avoidance behaviors (25, 26). Borrowing this logic, appetizing foods are normally positive, emotion-laden stimuli that activate the appetitive system and approach behaviors (21, 25, 27). Emotions evoked by UPF may automatically elicit high levels of approach motivation and influence individuals' eating behavior, including the motivation to consume UPF rather than unprocessed/minimally processed foods (UMPF). High exposure and easy access to UPF in different food environments, such as communities and organizations, may intensify their appeal to consumers, which affects consumer behavior (28–31). Thus, it is likely that emotions elicited by UPF available in food environments play a significant role in creating unhealthy and unsustainable diets.

Assessing emotional responses to foods is considered a valuable source of information about consumers' food preferences (12, 16, 32). Obtaining a valid and reliable measurement of food-evoked emotions is, however, a major concern when evaluating emotions evoked by food experiences, since emotions are difficult to verbalize (33). A well-established method for measuring emotional responses to affective pictures is the Self-Assessment Manikin scale, a non-verbal pictorial technique that makes the rating very intuitive, requiring little explanation (34, 35). In addition, reports of affective experiences using the Self-Assessment Manikin scale correlate with physiological responses (such as bradycardia or tachycardia, sweating, and contraction or relaxation of the facial muscles) to emotional stimuli providing, to some extent, information about implicit motivational responses (25, 36, 37).

Emotions can be studied from a dimensional perspective in which the diversity of emotions may be characterized by two main factors: hedonic valence (pleasantness: unpleasant vs. pleasant) and arousal (intensity of activation) (34). Pleasant affective responses are related to approach motivation, while unpleasant affective responses are related to avoidance motivation. Arousal affective responses reflect the level of motivational system activation (25, 26). The study of appetitive and defensive motivational systems can be accomplished through the presentation of normative emotional pictures during experimental investigations (34, 35). The Self-Assessment Manikin scale can be used (34) to assess the dimensions of pleasantness and arousal associated with each picture.

Understanding the emotional evocativeness of UPF may provide insights into the implementation of government policies to create healthier food environments. There is limited knowledge, however, about the emotional responses to UPF, categorized based on the NOVA food system classification (6, 7). In a previous study, we showed that the presentation of UPF pictures evoked strong emotional responses and approach motivation (21). However, it remains an open question whether UPF elicit higher levels of approach motivation than UMPF. In the present experimental study, we aimed to gain new insights into the emotional responses evoked by the presentation of UPF in comparison to UMPF. An experiment with undergraduate students was conducted that applied the Self-Assessment Manikin scale to evaluate the pleasantness and arousal ratings associated with pictures depicting UPF and UMPF. We hypothesized that participants would consider UPF pictures more pleasant and arousing than UMPF pictures. A secondary objective was to investigate whether emotional responses toward food stimuli were important triggers influencing the intention to consume UPF instead of UMPF. To this aim, we collected additional information about the participants' intention to consume the UMPF and UPF that were displayed. We hypothesized that the increased arousal and pleasantness ratings of UPF pictures compared to UMPF pictures would lead to a greater intention to consume UPF compared to UMPF.

## Materials and Methods

### Participants

The final sample consisted of 174 [144 women; mean (M) age = 20.7 years; standard deviation (SD) = 4.35] undergraduate students from health/biomedical sciences from Fluminense Federal University (Brazil) who met the following self-reported inclusion criteria: omnivorous, corrected-to-normal visual acuity and no eating disorder. The exclusion criteria were reporting prolonged fasting assessed by a hunger scale (38) before the experiment or not answering all the items in the questionnaires. The reported mean body mass index (BMI) of the participants was 21.7 kg/m^2^ (SD =6.2; BMI_Max_ = 41.4 kg/m^2^; BMI_Min_ = 14.5 kg/m^2^). According to criteria from the World Health Organization (39), 10% of the participants were classified with underweight, 69% with normal weight, 15% with overweight, and 6% with obesity. This anthropometric profile matched those from other studies with similar samples of Brazilian undergraduate students (40).

All the participants were students from health/biomedical sciences to minimize the individual variability in the sample regarding nutritional knowledge and eating habits (41, 42). The participants were naive to the purpose of the study. The local Research Ethics Committee approved the experiment, and all participants provided written informed consent before any experimental procedure was conducted. Data were collected between June 2018 and June 2019, before the COVID-19 outbreak.

### Study Exposure

In the present study the participants were exposed to a set of pictures varying in emotional content that served as stimuli to evoke emotions. The stimuli presented comprised food pictures (depicting UMPF or UPF) and pictures depicting other emotional content (such as nature, puppies, sports, adventure, erotic pictures, mutilated bodies, illness, loss, etc.). The latter were obtained from the International Affective Picture System (IAPS), a database designed to provide a standardized set of pictures for studying emotion (35). The focus here was on the appetitive motivation responses evoked by the presentation of food pictures. To this aim, we followed the normative rating procedure for the IAPS, a standard method proposed by Lang et al. (35), to establish the emotions elicited by each picture. The procedure consisted of exposing a group of participants to a set of pictures that may contain the pictures of interest (the food pictures in our case) in addition to pictures from other emotional categories as background. The use of pictures from the IAPS as background served as an affective basis for comparison during the evaluation of target pictures (food stimuli). Therefore, this method anchored the emotional classification scales and validated the values assigned to the target pictures (21, 35, 43, 44). This standard procedure provided between-group ratings, as previously noted (21, 25, 43, 45, 46). The details about the target pictures (food pictures) and the IAPS pictures used in the present study will be described in the next subsections.

#### Food Pictures

Sixty-four pictures depicting different types of food were divided into two groups based on the extent and purpose of food processing in accordance with NOVA system parameters (7). Thirty-two pictures presented UMPF, and the other 32 pictures presented UPF. Each group contained 16 pictures of foods with sweet taste and 16 pictures of foods with salty taste. The selected pictures of UMPF and UPF included foods that are part of the Brazilian population's diet (47). The UMPF group included pictures of fruits (e.g., apple, strawberry, watermelon, orange, kiwi, papaya, and peach), vegetables (e.g., lettuce, tomato, cooked broccoli, kale, cooked carrots, green beans, and cooked beans), nuts, home-cooked meals and grilled meat. These pictures were selected from the internet and the Open Library of Affective Foods (OLAF) (45). The UPF group included pictures of gums, chocolate disk, chocolate, soft drinks, cookies, breakfast cereal, ice cream, popsicles, jelly, hamburger, ready-to-eat pizza, ready-to-eat lasagne, nuggets, sausages, margarine, hotdogs, panettone with milk jam cream, Brazilian cheese bread, chocolate-covered marshmallow, bacon, microwave popcorn, cooked pork salami, chips, ready-to-drink chocolate milk and instant noodles. Pictures chosen for this group were selected from the internet or produced by a commercial food photographer. Those selected from the internet were in the public domain and free from copyright. All the foods (UPF and UMPF) were displayed as ready to be consumed by the participants, and they were presented unpackaged and cooked when pertinent. This procedure allowed better physical pairing between UPF and UMPF pictures. The pictures depicting UPF did not include the product's name or brand; thus, we focused on the intrinsic attributes of the products. The food pictures were also chosen to be used in conjunction with pictures from the IAPS, a well-known set of normative emotional pictures used for experimental investigations (35).

##### Classification of the Food Pictures Based on the NOVA System

To classify the food pictures as UPF and UMPF, we followed the criteria set out by the NOVA system (7). This system defines UPF as those that undergo industrial processing that convert whole foods into formulations of food substances, often modified by chemical substances containing additives and are high in calories, fat, sugar and/or salt (6, 7). UMPF are defined as foods in their natural form (consumed as found in nature) and foods that have undergone minimal processing (e.g., drying, grinding, vacuum packing, or non-alcoholic fermentation (6, 7). For these reasons, UMPF provide more fiber and micronutrients than UPF (48).

As the ultraprocessing of food typically entails, beyond the addition of chemical substances, an unbalanced nutritional composition, we estimated the ingredients and nutritional composition of each food depicted in the pictures. For the UMPF (see Supplementary Table 1), Brazilian food composition databases were assessed (49, 50). For the UPF (see Supplementary Table 2), samples of UPF were collected on the market, and the ingredients and nutritional composition were obtained from the ingredient list and the nutrition facts label on the package. These procedures were independently performed twice by two researchers to test the accuracy of the data. For the UPF, the brands were randomly chosen by each researcher.

To ascertain the nutritional quality of the foods (both UPF and UMPF) depicted in the pictures, the scores developed by the Food Standards Agency (FSA) (51) were used. Originally, the FSA scores were created to help food manufacturers calculate the nutritional quality of their products (mostly UPF). The FSA scores rank foods based on the nutrient content per 100 g of a food or beverage. The items energy (kJ), total sugar (g), saturated fatty acids (g) and sodium (mg) add points (0–10) to the final score, while the items fruits, vegetables and nuts (%), fiber (g) and protein (g) subtract points (0–5) from the final score. The final score can vary from −15 (very high nutritional quality) to +40 (very low nutritional quality). Based on the FSA score, a food is considered unhealthy when it scores four points or more, and for beverages, when it scores one point or more.

We also used the FSA scores to estimate the nutritional quality of UMPF. To calculate the points based on the percentage of fruits, vegetables or nuts, we used the value of 100% when fruits, vegetables, or nuts were themselves the foods depicted in the pictures. Two of the UMPF presented more than one type of food (tapioca filled with apple and honey and a homemade meal picture of a typical Brazilian lunch dish). For the tapioca, we used software (Dietbox^®^, https://dietbox.me/pt-BR) that estimates the weight of each food based on homemade measurements (i.e., apple slices). For the picture depicting a typical Brazilian lunch meal (beans, rice, red meat, lettuce, and beets), we weighed all the food types included in the meal before shooting the picture. The other FSA points based on the amount of energy, saturated fat, sugar, sodium, fiber, and protein were calculated based on Brazilian food composition databases (49, 50). Then, this information was applied to the formula proposed by the FSA (51) that combines the points based on the percentage of fruits, vegetables or nuts and the amount of energy, saturated fat, sugar, sodium, fiber and protein as previously described. Supplementary Tables 1, 2 (UMPF and UPF respectively) present the FSA scores calculated for each picture, as well as the ingredients and the energy, sugar, total fat, saturated fat, trans fat, sodium and fiber content per 100 g. The FSA scores independently estimated by the two researchers were highly correlated (rho = 0.95, *p* < 0.001).

The results obtained with the FSA scores showed that all the UPF scored far above the limit of 4 points for foods and 1 point for beverages (M_FSAscore_ = 15.13; SD = 6.85), which is considered poor nutritional quality (51). In contrast, all UMPF scored below these values and were considered healthy based on their FSA scores (M_FSAscore_ = −4.53; SD = 3.44). A *t*-test showed a large difference in nutritional quality between the food groups [*t*_62_ = −14.51, *p* < 0.001], confirming that the foods in the UPF group had worse nutritional quality than those in the UMPF group.

#### Pictures From the International Affective Picture System (IAPS)

In addition to the pictures of interest (food pictures), 74 pictures from the IAPS (35) depicting other content were also presented during the experiment. These pictures comprised distinct affective categories, including those typically rated as pleasant (nature, family, puppies, sports, adventure and erotic pictures), neutral (objects, people, and landscape) and unpleasant (pollution, illness, loss, accidents, contamination, attacking animals, attacking humans, and mutilated bodies). The food pictures were interspersed with the pictures from IAPS, the latter balanced in terms of emotional content (35). The neutral pictures of objects from the IAPS were used in the analyses to verify whether the selected food pictures (UPF and UMPF) were emotionally evocative, i.e., if they evoked greater emotional responses than neutral pictures, as previously described in the literature (21, 25, 45).

### Study Outcomes

The hedonic valence (pleasantness), arousal and “intention to consume” ratings were the outcome indicators of interest for the present study.

#### Hedonic Valence and Arousal

**Figure 1:**
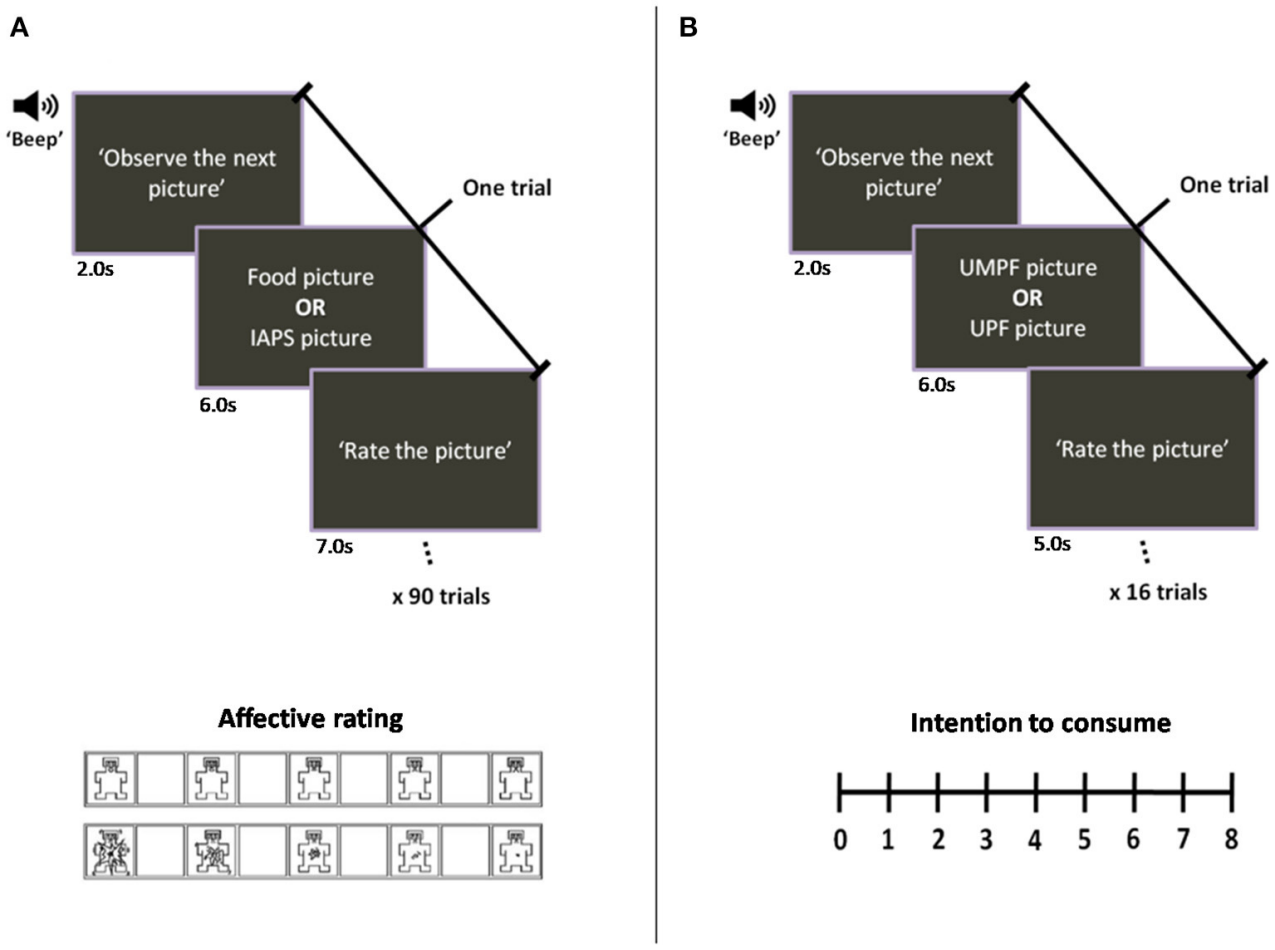
Schematic representation of the sequence of events in a trial. The experimental session was divided into two tasks. **(A)** Affective rating task: 16 food pictures (8 UPF and 8 UMPF) interspersed with 74 pictures in different affective categories from the IAPS were displayed, and the participants rated how they felt while viewing each picture by marking the Self-Assessment Manikin scale. **(B)** After the affective rating task, participants performed a second task in which they rated their “intention to consume” each of the 16 foods presented in the pictures on a scale ranging from 0 (none) to 8 (maximum). UMPF, unprocessed/minimally processed foods; UPF, ultra-processed foods.

The pictures were evaluated using the paper-and-pencil version of the Self-Assessment Manikin scale, a technique that indicates the level of emotional evocativeness of each picture (34). For each dimension (pleasantness and arousal), there is a row of five figures interleaved by blank spaces, yielding nine intensity levels. For the pleasantness dimension, the manikins exhibit expressions that range from “smiling-happy” (score = 9) to “frowning-unhappy” (score = 1). For the emotional arousal dimension, the expressions of the manikins range from an “excited wide-eyed” figure (score = 9) to a “relaxed-sleepy” figure (score = 1) (see Figure 1A, bottom panel). Lang et al. developed the Self-Assessment Manikin scale to create a set of standard pictures (the IAPS) calibrated for affective responses. The methodology used in the IAPS generates standard picture sets that evoke similar emotional responses across individuals and groups (35).

#### Intention to Consume

The participants also rated their “intention to consume” the foods depicted in the pictures. This procedure was conducted to understand whether the emotion elicited by the food pictures was related to the participants' intention to consume the presented food. After the emotional rating session, in which the target food pictures and the IAPS pictures were presented, we repeated the presentation of the target food pictures without the pictures from IAPS (i.e., eight pictures of UMPF and eight pictures of UPF were presented). This rating was made on a 9-point Likert-type scale (0 = none, 8 = maximum) (21).

### Hunger Assessment

We used a hunger scale (38) to estimate the level of subjective hunger of the participants immediately before the experimental session. The scale consisted of the following hunger-related items: time since last eating, subjective hunger, estimate of the amount of their favorite food that they could eat, and estimate of the time until the next expected meal. A total score was computed combining the hunger items as previously suggested by Tapper and Turner (52). Hunger was assessed to evaluate its possible influence on the participants' affective ratings.

### Experiment Description

#### Apparatus

The ratings sessions were conducted in a dimly lit room with desks placed in rows in front of a slide projection screen. The desks were arranged in such a manner that the screen was perfectly visible to every participant. The stimuli were displayed with a projector connected to a microcomputer running the software E-prime 3.0 Professional (Psychology Software Tools, Inc., Sharpsburg, PA, USA), which controlled the timing of stimuli presentations.

#### Procedure

The experiment consisted of a rating session divided into two tasks. During the first task, the participants viewed the target food pictures interspersed with pictures from the IAPS and then rated the valence and arousal of each picture. During the second task, the participants were exposed to the target food pictures again (without the IAPS pictures) and rated each picture based on their intention to consume the foods depicted in them (Figure 1). To minimize fatigue over time, we limited the number of target pictures presented to each participant, as is usual in the IAPS normative rating procedure (35). Each rating session included only 16 (instead of 64) food target pictures: eight UMPF pictures and eight UPF pictures, with each food group containing four sweet and four savory food pictures. To complete the assessment of the 32 UMPF, 32 UPF and 32 pictures from the IAPS, we assembled four sets of pictures that varied across sessions. These picture sets were presented in different evaluation sessions and rated by different participants. Each set of pictures received ratings from a maximum of 46 and a minimum of 41 participants. The four picture sets were matched in terms of physical and aesthetic properties (see Methods section Control of Physical and Aesthetic Properties of Pictures for more details).

During the first part of the session (affective rating task), the food pictures (16 in total) and the eight pictures of objects from the IAPS (neutral category) were presented in addition to 66 emotional background pictures from the IAPS (i.e., pictures other than the pictures of neutral objects). These pictures from the IAPS did not vary across sessions, as occurred with the food pictures and pictures of objects from the IAPS. During the second part of the session (“intention to consume” rating task), the 16 food pictures presented during the first part were presented again (without the other pictures); thus, they also varied across sessions.

The sequence of events during the session occurred as follows. Upon arrival, the participants were asked to sit and to read and sign the consent form if they were willing to participate in the study. Each participant then received a copy of the instructions and a rating booklet. Before starting the affective rating task (Figure 1A), the participants filled out the hunger scale (38) and then watched a didactic video explaining the upcoming task. Training was initially performed using nine extra pictures from the IAPS from diverse affective categories. After the training, the first part of the session was performed, which included 90 trials involving 74 IAPS pictures (including eight pictures of objects), eight UMPF pictures, and eight UPF pictures. Each trial began with a brief “beep” (50 ms) associated with a preparation slide (“Observe the next picture”) that lasted 2 s. Then, the picture was presented for 6 s to be appraised. During the next 7 s, the participants were asked to estimate how they felt about the picture by rating it along the dimensions of hedonic valence (pleasantness) and arousal using the paper-and-pencil version of the Self-Assessment Manikin scale (34) (Figure 1A). The sequential order of the presented pictures was pseudorandomized, with the constraint that the affective category (neutral, pleasant or unpleasant) could not be repeated more than three consecutive times.

After the affective classification of the pictures, a new didactic video was displayed, which explained the task in which participants assigned ratings of “intention to consume” each food represented in the pictures. After performing a training block containing six extra UMPF and UPF pictures, the participants rated their “intention to consume” each of the 16 foods depicted in the pictures (8 UMPF and 8 UPF displayed in a random order) presented during the previous affective classification task. Each trial consisted of a brief “beep” (50 ms) associated with the presentation of a preparation slide (“Observe the next picture”) that lasted 2s, followed by the presentation of the food picture that lasted 6s. Finally, the classification slide (“Rate the picture”) was presented for 5s, in which the participants rated their “intention to consume” the food represented in the picture (Figure 1B). Ultimately, the participants filled out a form with their personal data.

#### Control of Physical and Aesthetic Properties of Pictures

The pictures of UPF, UMPF and neutral objects were all matched by complexity (clear figure-ground pictures compared with complex scenes), perceptual properties (brightness, contrast, and spatial frequency) and aesthetics across the four sets of pictures. This procedure was conducted because it has been shown that picture complexity, perceptual properties and aesthetics rather than emotionality may be responsible for some of the differences in neural responses and subjective ratings to neutral and emotional pictures (53–55).

Brightness, contrast and spatial frequency were defined in accordance with Bradley et al. (53). We used an average RGB (red, green, blue) value for each pixel, averaged over all pixels, to define brightness. The standard deviation of the mean RGB values was computed in pixels for each column and the standard deviation of these was used as an index to calculate the contrast. To ascertain the spatial frequency, the frequency of the median fast Fourier transform power was determined for each row and column and then averaged. Picture complexity was extracted from a compressed file size measure since JPEG file compression is correlated with subjective complexity ratings in affective pictures (56). The aesthetic quality of the pictures was calculated *via* the Aesthetic Quality Inference Engine (ACQUINE) system (57), which is a machine learning-based system that is publicly accessible online for the prediction of the aesthetic quality of pictures. The software extracts visual features from the pictures and performs classification and prediction through a support vector machine-based classifier.

We conducted repeated-measures ANOVAs with *picture category* (3 levels: UMPF, UPF or neutral objects) as the within-subjects variable and *picture set* (four levels) as the between-subjects variable. We succeeded in controlling the physical and aesthetic properties of the pictures since ANOVAs did not reveal significant main effects or interaction effects for any of the variables. The *picture category* main effect results were as follows: brightness [*F*_(2, 56)_ = 0.04, *p* = 0.96], contrast [*F*_(2, 56)_ = 1.81, *p* = 0.17], spatial frequency [*F*_(2, 56)_ = 0.08, *p* = 0.92], complexity [*F*_(2, 56)_ = 0.23, *p* = 0.79], and aesthetics [*F*_(2, 56)_ = 1.02, *p* = 0.37]. The interaction between *picture category* and *picture set* results were as follows: brightness [*F*_(6, 56)_ = 0.11, *p* = 0.99], contrast [*F*_(6, 56)_ = 0.27, *p* = 0.95], spatial frequency [*F*_(6, 56)_ = 0.81, *p* = 0.57], complexity [*F*_(6, 56)_ = 0.86, *p* = 0.53], and aesthetics [*F*_(6, 56)_ = 2.08, *p* = 0.07].

### Data Analysis

#### Distribution of the Affective Ratings in the Bidimensional Affective Space of Hedonic Valence and Arousal

The ratings of hedonic valence (pleasantness) and arousal of a heterogeneous emotion-laden group of pictures plotted in a Cartesian plane are disposed in vectors that point in two directions and represented by a “boomerang” shape (25). The upper arm of the boomerang indexes appetitive (approach-like) motivation, and the lower arm indexes defensive (avoidance-like) motivation. A first step was performing this analysis to verify whether the rating distribution in the bidimensional (valence-arousal) affective space in our experiment fits the typical boomerang shape reported by Lang and colleagues in successive North American versions of the IAPS (34, 35). For each picture, we calculated the average of the rates and standard deviations for pleasantness and arousal attributed by the participants in each picture set (minimum of 41 and maximum of 46 participants per picture). Thus, the ratings from different participants were combined to obtain a rating score per picture. Then, these rating scores obtained for each picture were plotted in a Cartesian plane that had pleasantness on the x-axis and arousal on the y-axis. In addition, we used a Spearman's correlation test to confirm the similarity between the ratings of IAPS pictures obtained in our sample and those reported for North Americans (35). To facilitate visualization in the Cartesian plane, the ratings along the hedonic valence (pleasantness) dimension were converted to numbers ranging from −4 (extremely unpleasant) to +4 (extremely pleasant). Therefore, the most negative values are in the defensive space (in the lower arm of the boomerang) and the most positive values are in the appetitive space (in the upper arm of the boomerang).

#### Food Pictures vs. Neutral Pictures

Since the neutral picture data violated the assumption of a normal distribution (*p* < 0.05), as assessed by the Kolmogorov–Smirnov test, we conducted a Wilcoxon test to compare the participants' affective ratings to food pictures (the average of the ratings for the UPF and UMPF) and neutral pictures. This analysis was performed to confirm whether the food pictures (UPF and UMPF) were more pleasant and arousing than the pictures of neutral objects from the IAPS, and thus being positioned in the upper arm of the boomerang (assumed to reflect the appetitive motivational system), as previously described (21, 25, 35, 45). For this and the next analyses, the affective ratings from the different groups of food pictures (i.e., UPF and UMPF) were combined to obtain a mean rating value per participant. The reader can find the mean pleasantness and arousal ratings for each participant in the Supplementary material (see Supplementary Table 3). We performed two separate analyses: one for pleasantness ratings and the other for arousal ratings. The *p*-value considered for significance was *p* < 0.05.

#### Ultra-Processed Foods vs. Unprocessed/Minimally Processed Foods

As the pictures that comprised each picture set were rated by different participants, we conducted repeated-measures ANOVA with the *NOVA group* (UPF or UMPF) as a within-subjects variable and *picture set* (four levels) as a between-subjects variable. This analysis was performed since the data did not violate the assumption of normal distribution (*p* ≥ 0.05), as assessed by the Kolmogorov–Smirnov test. The *p*-value considered for significance was *p* < 0.05. We performed two separate ANOVAs: one for pleasantness ratings and another for arousal ratings. These analyses were performed to determine whether there was a significant difference between the pleasantness and arousal mean ratings of the pictures depicting UPF and those depicting UMPF and possible interactions with the picture sets.

We also performed analysis of covariance (ANCOVA) to evaluate the effects of the *NOVA group* (UPF or UMPF) on the affective ratings (pleasantness and arousal) while including hunger as a covariate.

#### Affective Ratings and Intention to Consume

We used a paired Wilcoxon signed rank test to compare the intention to consume mean ratings obtained for pictures depicting UMPF and UPF. To test whether the greater the affective ratings to UPF relative to UMPF indicated the greater the intention to consume UPF relative to UMPF, we created an index of emotional reactivity to UPF vs. UMPF. For this purpose, we subtracted the affective rating values obtained for the UMPF from the affective ratings obtained for the UPF (emotional reactivity index = UPF minus UMPF). The same index was calculated for the “intention to consume” ratings. The “emotional reactivity” and “intention to consume” indexes for each participant are presented in the Supplementary material (see Supplementary Tables 3, 4). Spearman's correlations were used to verify the relationship between the emotional reactivity indexes obtained from pleasantness and arousal ratings and the index obtained from the “intention to consume” ratings. The normality assumption was violated for both emotional reactivity and intention to consume indexes (*p* < 0.05, Kolmogorov–Smirnov test). The *p*-value considered for significance was *p* < 0.05.

## Results

### Distribution of the Affective Ratings in the Bidimensional Affective Space of Pleasantness and Arousal

**Figure 2:**
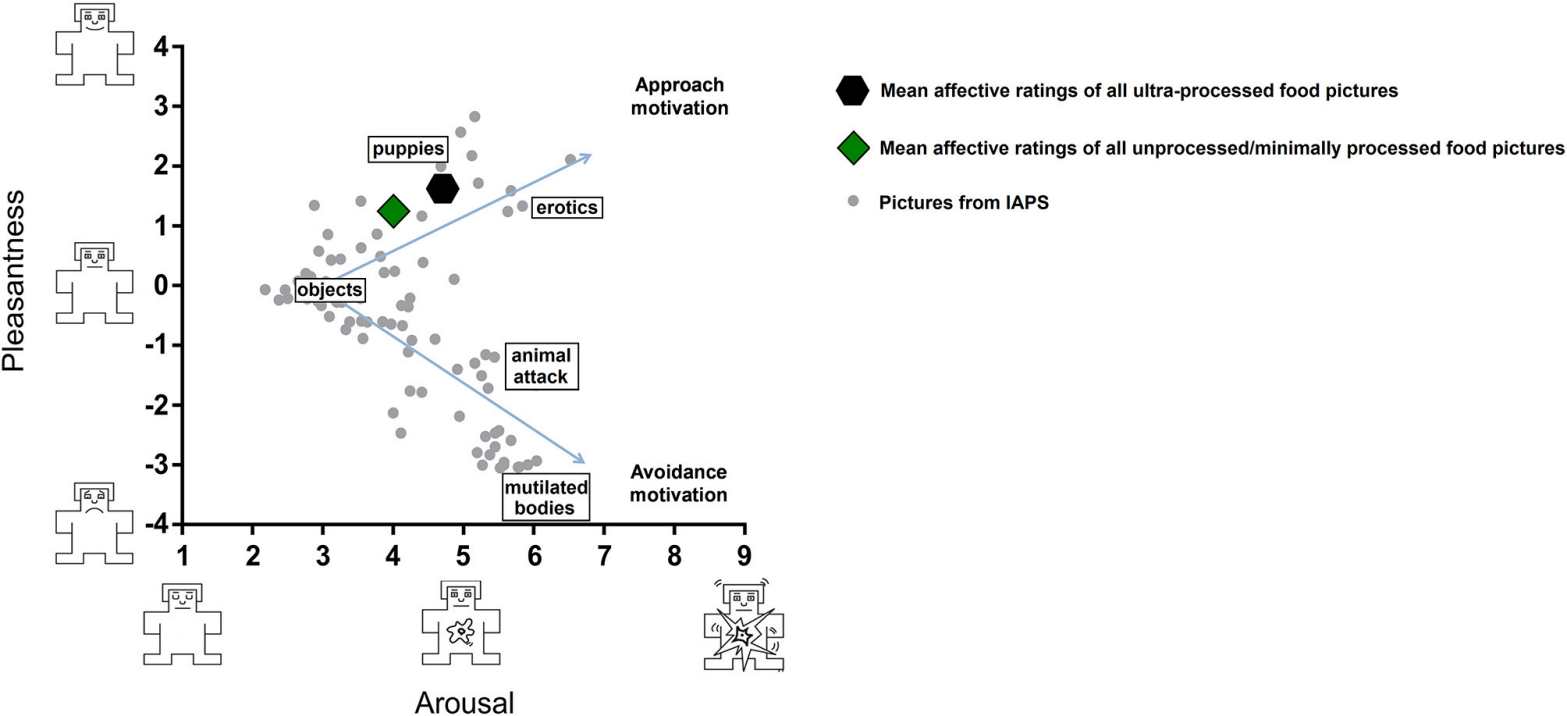
Affective space. Illustration of the bidimensional affective space defined by each picture's mean pleasure (y-axis) and arousal (x-axis) ratings with presentation of the motivational tendencies of approach (top half of plot) and avoidance (bottom half of plot) elicited by each picture. Each gray dot (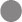) in the plot represents IAPS pictures used as background (e.g., nature, family, sports, adventure, erotic pictures, puppies, people, landscape, objects, pollution, illness, loss, accidents, contamination, attacking animals, attacking humans, and mutilated bodies). The mean affective ratings of all UPF pictures are represented by the hexagon symbol (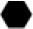) and mean of all UMPF pictures by the diamond symbol (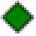). IAPS, International Affective Picture System; UMPF, unprocessed/minimally processed foods; UPF, ultra-processed foods.

Figure 2 shows the distribution of the picture ratings in the bidimensional affective space defined by the pleasantness (x-axis) and arousal (y-axis) dimensions. The results showed that the IAPS pictures (gray dots) were properly distributed in the affective space, showing the typical boomerang-shaped distribution observed in previous studies (21, 25, 43–45). The pleasant pictures from the IAPS (e.g., erotic pictures, sports, families, nature, and puppies) were located in the upper half of the chart, while unpleasant pictures from the IAPS (e.g., mutilation, illness, pollution, accidents, and disgusting pictures) were located in the lower half of the chart. The neutral pictures from the IAPS (objects) were located in the center of the graph. We found a high correlation between the ratings of IAPS pictures obtained in our sample and those reported for North Americans (35) in both the pleasantness (rho = 0.95, *p* < 0.001) and arousal (rho = 0.89, *p* < 0.001) dimensions. Thus, we found the expected pattern of distribution of IAPS pictures in the affective space, ratifying that we correctly applied the methodology proposed by Lang et al. (35).

**Figure 3:**
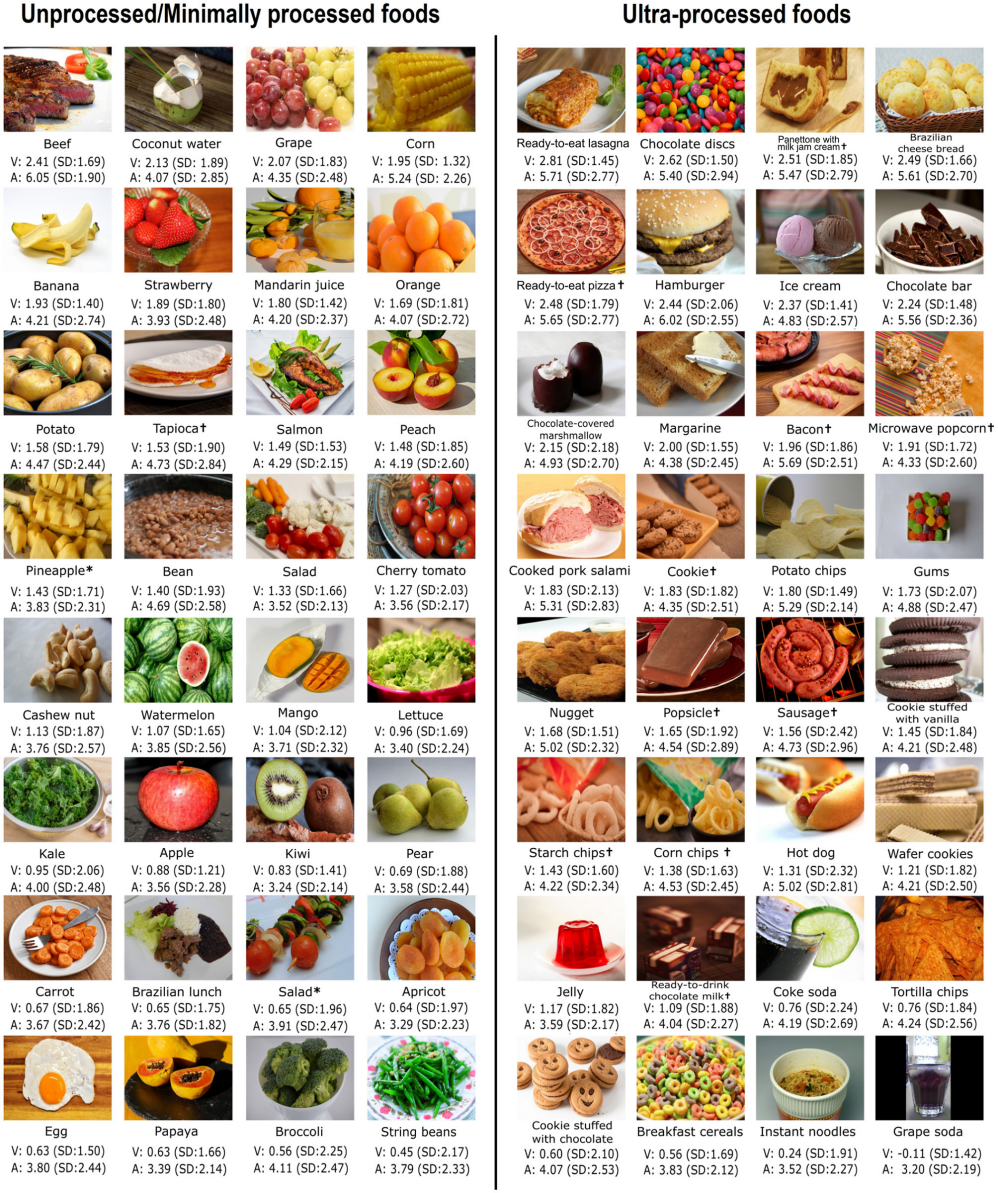
Pictures of the UMPF and UPF presented in the study. The mean and SD values of pleasantness (hedonic valence; V) and arousal (A) are indicated below each picture. Pleasantness ratings ranged from −4 (extremely unpleasant) to +4 (extremely pleasant) with 0 indicating neutrality. Arousal ratings ranged from 1 (very low arousal) to 9 (extremely arousing). The pictures with asterisks in the UMPF group (fru_5772–pineapple and veg_0092–salad) belong to the Open Library of Affective Foods (45). Pictures with the symbol ^†^ were produced by the commercial photographer Alexander Chiacchio. UMPF, unprocessed/minimally processed foods; UPF, ultra-processed foods; SD, standard deviation.

Food pictures (UPF and UMPF; hexagon symbol and diamond symbol, respectively) were located in the upper half of the chart (in the appetitive space), as were the pleasant pictures from the IAPS. The pleasantness and arousal ratings seemed to diminish from UPF (hexagon symbol) to UMPF (diamond symbol). Notably, some pictures of UPF (such as hamburgers, ready-to-eat lasagna, ready-to-eat pizza, chocolate bars, panettone with milk jam cream, and chocolate disk) were classified as extremely arousing and pleasant. These UPF pictures were positioned at the upper end of the appetitive arm in the affective space, next to emotional picture categories that strongly activated appetitive motivation in university students, such as pictures depicting erotica (25). The mean pleasantness and arousal ratings for each food picture (64 in total) are presented in Figure 3.

### Food Pictures vs. Neutral Pictures

As shown in Figure 2, the pictures depicting foods are positioned in the upper arm of the boomerang-shaped affective space, which is assumed to reflect the appetitive motivational system. Our analyses confirmed that the food pictures were rated as more pleasant (M_food_ _pictures_ = 1.43, SD = 0.88; M_neutral_ _pictures_ = −0.01, SD = 0.66; *Z* = 10.84, *p* < 0.001) and more arousing (M_food_ _pictures_ = 4.35, SD = 1.66; M_neutral_ _pictures_ = 3.19, SD = 1.44; *Z* = 8.19, *p* < 0.001) than the neutral pictures. Thus, the food pictures in our study were effective in triggering the appetitive motivational system as previously described (21, 35, 45).

### Ultra-Processed Foods Pictures vs. Unprocessed/Minimally Processed Foods Pictures

Repeated-measures ANOVA revealed a main effect of *NOVA group* for pleasantness ratings [*F*_(1, 170)_ = 17.05, *p* < 0.001] and for arousal ratings [*F*_(1, 170)_ = 46.48, *p* < 0.001]. The UPF were ranked as more pleasant and more arousing than the UMPF (Table 1). Therefore, UPF evoked greater emotional responses than UMPF.

**Table 1:** Mean pleasantness and arousal ratings for the pictures depicting UMPF and UPF.

	**UMPF**		**UPF**	
	**Mean**	**SD**	**Mean**	**SD**
Pleasantness	1.24^a^	1.07	1.62^b^	1.06
Arousal	4.00^a^	1.77	4.70^b^	1.82

*Pleasantness ratings could range from −4 (extremely unpleasant) to +4 (extremely pleasant) with 0 indicating neutrality. Arousal ratings could range from 1 (very low arousal) to 9 (extremely arousing). Mean rating scores within a row with different superscript letters are significantly different (p < 0.05). SD, standard deviation; UMPF, unprocessed/minimally processed foods; UPF, ultra-processed foods*.

The main effect of *picture set* and the interaction between *picture set* and *NOVA group* were not significant for either pleasantness [*F*_(3, 170)_ = 0.79, *p* = 0.50] or arousal [*F*_(3, 170)_ = 0.30, *p* = 0.83] ratings. Therefore, the different picture sets did not affect the difference between affective ratings to pictures of UMPF and UPF.

We also performed ANCOVA to test the impact of hunger on affective ratings to pictures of the UMPF and UPF. The difference between the UPF and UMPF was maintained after the inclusion of hunger as a covariate for both pleasantness ratings [*F*_(1, 345)_= 10.86, *p* < 0.05] and arousal ratings [*F*_(1, 345)_ = 13.46, *p* < 0.05].

### Affective Ratings and Intention to Consume

**Figure 4:**
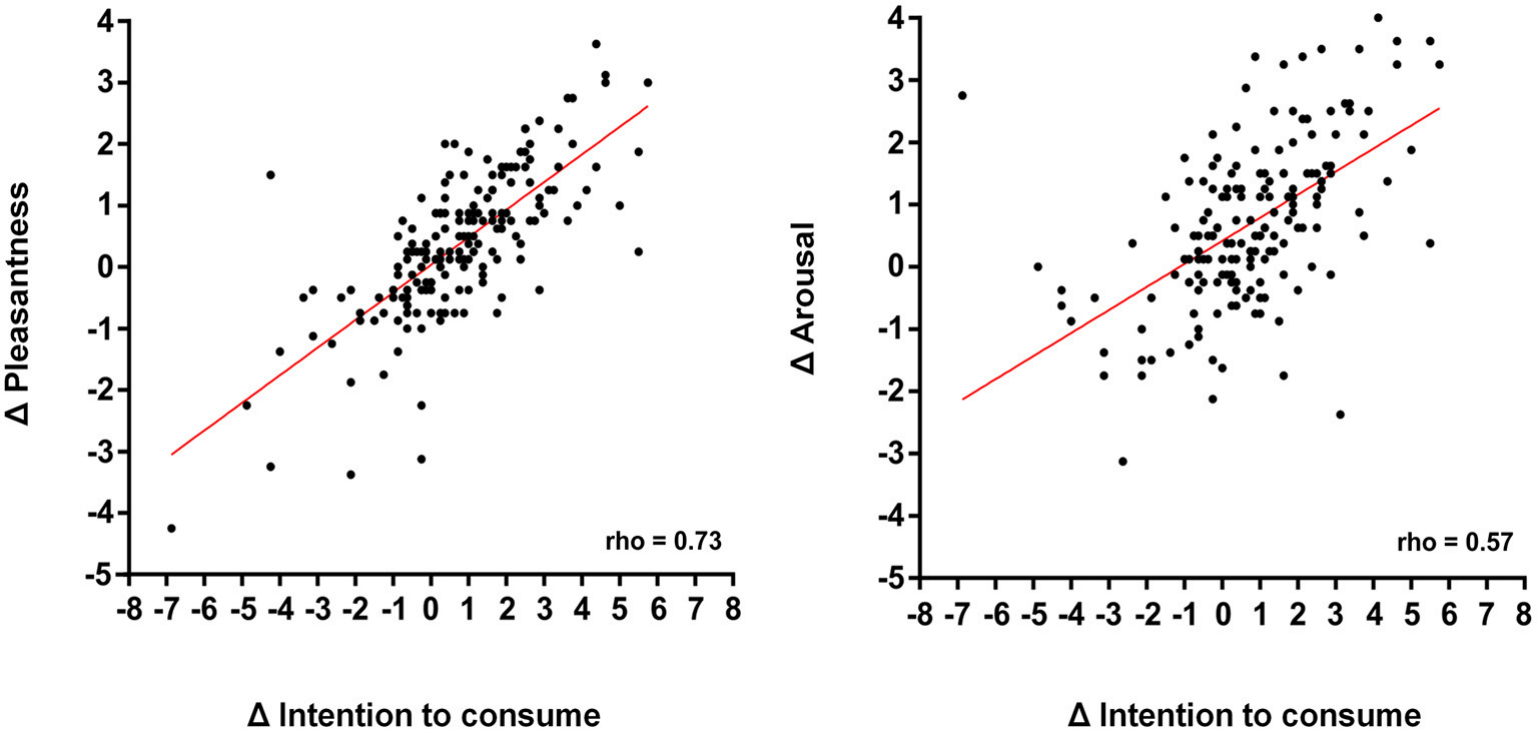
Correlations between the emotional reactivity index obtained from pleasantness (left panel) and arousal (right panel) ratings and the index obtained from the “intention to consume” ratings. The index was calculated by subtracting the mean ratings assigned for the UMPF from the mean ratings assigned for the UPF (delta = UPF minus UMPF). UMPF, unprocessed/minimally processed foods; UPF, ultra-processed foods.

The pictures depicting UPF (M = 4.56, SD = 1.72) received higher “intention to consume” ratings than the pictures depicting UMPF (M = 3.80, SD = 1.82; *Z* = 5.35, *p* < 0.001). An interesting question was whether this higher intention to consume UPF, relative to that for UMPF, was related to the emotional reactivity evoked by these pictures. To answer this question, we used the index (delta = UPF minus UMPF) that was created for the affective ratings and for the “intention to consume” ratings to conduct a correlation analysis between these indexes. There was a positive correlation between “intention to consume” and “emotional reactivity” indexes for both pleasantness (rho = 0.73, *p* < 0.001) and arousal (rho = 0.57, *p* < 0.001) ratings (Figure 4). Thus, the higher the affective ratings (pleasantness and arousal) assigned to the UPF were, the higher the intention to consume them when compared to UMPF.

## Discussion

### Main Findings

In agreement with previous evidence (21), UPF were perceived as highly pleasant and arousing, being positioned in the upper arm of the boomerang-shaped affective space that represents appetitive/approach motivation. Notably, UPF received higher affective and “intention to consume” ratings than UMPF. Additionally, the affective responses correlated with a higher intention to consume UPF than UMPF. Thus, UPF were perceived as more motivationally appetitive than UMPF, which paralleled a higher intention to consume them compared to UMPF.

UPF are associated with aggressive marketing strategies and hyperpalatability that may make them more attractive than UMPF. UPF are designed to be hyperpalatable, which enhances their hedonic value and the pleasure associated with their consumption (58). UPF usually contain a combination of calorie-dense ingredients (e.g., sucrose and saturated fat) that are not typically found combined in nature and that are very pleasing to the human palate. Additives added to UPF also give them intense sensory properties, turning them into very attractive foods (6, 59, 60). Since flavor is a multisensory experience (61), UPF sensory properties stimulate other human senses in addition to taste, such as vision, smell and somatosensation. For instance, additives may artificially recreate scents of fruits or emphasize colors, and frying oils provide crispy textures (62, 63). In summary, sensory properties of UPF may make it very difficult to resist the overwhelming temptation to consume them, a factor exacerbated by aggressive marketing campaigns (64, 65). Thus, it is possible that our results reflect the ability of visual cues associated with UPF to stir consumers' emotions and elicit approach motivation, with an impact on consumers' intention to consume UPF rather than UMPF.

### Relevance to Food Policy

In view of the emotional effects on consumer behavior (12, 13), policy makers may want to consider evidence on emotional responses to UPF and UMPF when developing and testing public health strategies to promote healthy and sustainable food environments. Food environments include the physical spaces where consumers engage with food systems to make decisions about acquiring and consuming UPF and UMPF (11, 66, 67). UPF may implicitly activate high approach motivation and drive consumers to choose UPF over UMPF in those spaces. Thus, regulatory measures impacting food environments, such as the restriction of marketing, availability and affordability to UPF as well as the adoption of front-of-pack nutrition labels, should be aligned with evidence on emotional evocativeness of UPF and UMPF. This evidence may help to support food policies aiming to minimize the attractiveness and consumption of UPF and increase the attractiveness and consumption of UMPF.

### Reliability of the Affective Ratings Obtained in the Present Study

The reliability and validity of the affective ratings obtained with the food pictures in our study is supported by the replication of findings from emotion studies that also applied the normative rating procedure for pictures in the IAPS. First, we replicated the typical boomerang-shaped distribution of picture ratings within the bidimensional (valence-arousal) affective space, which has been consistently reported in emotion studies (21, 25, 45, 68, 69). In agreement with previous emotion studies, the food pictures in our study activated the appetitive motivational system since their mean rating scores were located in the upper arm of the boomerang and differed from the ratings obtained with pictures of neutral objects (21, 25, 45). Additionally, the mean affective ratings obtained in our study for food pictures from the OLAF were similar to the mean ratings found in the original study conducted with university students from Spain (45). Finally, the affective ratings for the pictures from the IAPS obtained in the present study were highly correlated with the affective ratings obtained in the original IAPS study (35).

In summary, the normative rating procedure for the IAPS (35) applied here provided (A) a valid measure of emotional evocativeness per food picture that can be extrapolated to other individuals and groups and (B) a normative set of UPF and UMPF pictures that can be used in future studies.

### Limitations

The present study has some limitations. First, the sample consisted of university students. A university student sample was chosen to enable the comparison with previous emotion studies that employed the normative rating procedure for the IAPS (21, 25, 35, 45, 69, 70). Second, the sample was composed predominantly of women. This limitation is explained by the higher number of women among students from health/biomedical undergraduate courses in Brazil (40). However, it is important to highlight that UPF evoked greater positive emotions than UMPF even in a sample of health/biomedical students, who usually show higher levels of nutritional knowledge and healthier eating habits than students in other programs (41, 42). In other words, a food environment saturated with UPF may lead consumers to approach them, even when they have the knowledge to make healthier food choices. Although the sample choice was pertinent in the present study, studies using more diverse populations would be interesting in the future.

Finally, we focused on the intrinsic attributes of the foods by presenting the foods unpackaged (in the case of UPF) and ready to consume. Although the better pairing between UPF and UMPF pictures provided by this procedure can be considered an advantage, the results should be interpreted with caution with regard to UPF inside packaging. Future studies could explore extrinsic UPF attributes such as packaging, advertising, and branding.

## Conclusion

Our results contribute to understanding the emotional processing of UPF and UMPF as visual cues with a possible impact on the intention to consume UPF over UMPF. The present study sheds new light on the role of UPF-evoked emotions in contributing to unhealthy food environments and may help in the development of policies aiming to promote healthy and sustainable food systems to curb the Global Syndemic.

## Data Availability Statement

The datasets presented in this study can be found in Lemos and David (71).

## Ethics Statement

The studies involving human participants were reviewed and approved by Research Ethics Committee of the Hospital Universitário Antonio Pedro of the Universidade Federal Fluminense. The patients/participants provided their written informed consent to participate in this study.

## Author Contributions

ID, TL, GC, RC, LO, and MP conceptualized and designed the experiments. ID, TL, GC, and JS analyzed the data. ID, TL, LO, MP, GS, BM, DC, and NK drafted the manuscript. TL, GC, RC, LS, and JS collected the data. ID, RC, LO, and MP contributed to the analysis tools and materials. All authors reviewed draft versions of the manuscript for salient intellectual content and provided suggestions and critical feedback, read, and approved the final manuscript.

## Funding

This study was supported by the following Brazilian research agencies: Coordination for the Improvement of Higher Education Personnel, Brazil (CAPES)–Financing code 001, National Council for Scientific and Technological Development (CNPQ), and Carlos Chagas Filho Foundation for Supporting Research in the State of Rio de Janeiro (FAPERJ).

## Conflict of Interest

The authors declare that the research was conducted in the absence of any commercial or financial relationships that could be construed as a potential conflict of interest.

## Publisher's Note

All claims expressed in this article are solely those of the authors and do not necessarily represent those of their affiliated organizations, or those of the publisher, the editors and the reviewers. Any product that may be evaluated in this article, or claim that may be made by its manufacturer, is not guaranteed or endorsed by the publisher.
