# Supplementary material for: Ultra-Processed Foods Elicit Higher Approach Motivation Than Unprocessed and Minimally Processed Foods
Source: Front Public Health. 2022 Jun 21;10:891546. doi: 10.3389/fpubh.2022.891546 (PMC9253546; doi:10.3389/fpubh.2022.891546)
Supplement: Supplementary file 1 [file Table_1.DOCX]

**Table S1.** Nutritional content and ingredients of the unprocessed and minimally processed foods presented in the experiment.

| **Unprocessed/minimally processed foods** | | | | | | | | | |
| --- | --- | --- | --- | --- | --- | --- | --- | --- | --- |
| **Food** | **Ingredients** | **Energy (kJ)*** | **Sugar (g)*†** | **Total fat (g)*** | **Saturated fat**  **(g)*** | **Trans fat (g)*** | **Sodium**  **(mg)*** | **Fiber (g)*** | **FSA**  **score** |
| beef | beef | 996.80 | 0.00 | 11.33 | 4.50 | 0.00 | 60.66 | 0.00 | 1 |
| coconut water | coconut water | 80.55 | 1.49 | 0.20 | 0.18 | 0.00 | 106.52 | 1.12 | -5 |
| grape | grape | 205.07 | 15.48 | 0.16 | 0.00 | 0.00 | 7.92 | 0.93 | -3 |
| corn | corn | 669.39 | 3.16 | 7.18 | 1.12 | 0.00 | 244.96 | 4.25 | -8 |
| banana | banana | 411.08 | 12.23 | 0.07 | 0.00 | 0.00 | 1.00 | 2.04 | -4 |
| strawberry | strawberry | 126.03 | 4.90 | 0.31 | 0.00 | 0.00 | 0.00 | 1.72 | -6 |
| mandarin juice | mandarin orange | 150.93 | 14.28 | 0.00 | 0.00 | 0.00 | 0.00 | 0.00 | -2 |
| orange | orange | 190.11 | 8.37 | 0.10 | 0.00 | 0.00 | 0.00 | 1.12 | -5 |
| potato | potato | 215.65 | 0.86 | 0.00 | 0.00 | 0.00 | 2.29 | 1.34 | -1 |
| tapioca | tapioca, apple, and honey | 604.15 | 12.41 | 0.12 | 0.02 | 0.00 | 1.33 | 1.61 | -1 |
| salmon | salmon | 956.10 | 0.00 | 14.04 | 3.14 | 0.00 | 85.14 | 0.00 | 0 |
| peach | peach | 151.99 | 8.38 | 0.00 | 0.00 | 0.00 | 0.00 | 1.42 | -6 |
| pineapple | pineapple | 201.98 | 9.26 | 0.12 | 0.00 | 0.00 | 0.00 | 0.99 | -4 |
| bean | bean | 319.76 | 0.30 | 0.54 | 0.10 | 0.00 | 1.76 | 8.51 | -7 |
| salad | grape tomatoes, cauliflower, baby carrots, and broccoli | 111.65 | 0.47 | 0.35 | 0.06 | 0.00 | 26.71 | 2.33 | -8 |
| cherry tomato | cherry tomato | 87.78 | 2.62 | 0.33 | 0.05 | 0.00 | 9.00 | 1.03 | -6 |
| cashew nut | cashew nut | 2399.32 | 6.20 | 46.35 | 9.16 | 0.00 | 16.00 | 3.00 | 3 |
| watermelon | watermelon | 136.30 | 6.21 | 0.00 | 0.00 | 0.00 | 0.00 | 0.12 | -4 |
| mango | mango | 211.88 | 12.73 | 0.22 | 0.10 | 0.00 | 0.00 | 2.07 | -5 |
| lettuce | lettuce | 36.74 | 0.79 | 0.13 | 0.00 | 0.00 | 7.31 | 1.02 | -6 |
| kale | kale | 113.21 | 1.41 | 0.55 | 0.12 | 0.00 | 6.17 | 3.12 | -10 |
| apple | apple | 232.03 | 10.40 | 0.00 | 0.00 | 0.00 | 0.00 | 1.35 | -4 |
| kiwi | kiwi | 213.75 | 8.97 | 0.63 | 0.06 | 0.00 | 0.00 | 2.65 | -7 |
| pear | pear | 222.84 | 9.79 | 0.11 | 0.00 | 0.00 | 0.00 | 3.01 | -7 |
| carrot | carrot | 142.82 | 4.73 | 0.17 | 0.00 | 0.00 | 3.33 | 3.18 | -8 |
| Brazilian lunch | beef, rice, beans, lettuce, beets, garlic, onions, bay leaves, broth, wood wine, butter, wheat flour, and salt | 554.85 | 0.30 | 3.94 | 1.55 | 0.00 | 156.46 | 1.57 | -4 |
| salad | cherry tomatoes, eggplant, zucchini, peppers, and onions | 99.24 | 1.94 | 0.29 | 0.05 | 0.00 | 1.63 | 1.60 | -7 |
| apricot | apricot | 1007.38 | 53.00 | 0.50 | 0.00 | 0.00 | 10.00 | 7.00 | 1 |
| egg | egg | 1003.98 | 1.22 | 18.59 | 4.12 | 0.00 | 166.11 | 0.00 | 2 |
| papaya | papaya | 167.87 | 4.86 | 0.12 | 0.00 | 0.00 | 1.63 | 1.04 | -5 |
| broccoli | broccoli | 103.00 | 1.31 | 0.46 | 0.10 | 0.00 | 2.12 | 3.42 | -10 |
| string beans | string beans | 104.17 | 2.30 | 0.17 | 0.00 | 0.00 | 0.00 | 2.38 | -9 |

FSA, Food Standards Agency.

*Per 100 g or 100 mL.

† Considered the values of saccharides naturally present in foods
