# Supplementary material for: Ultra-Processed Foods Elicit Higher Approach Motivation Than Unprocessed and Minimally Processed Foods
Source: Front Public Health. 2022 Jun 21;10:891546. doi: 10.3389/fpubh.2022.891546 (PMC9253546; doi:10.3389/fpubh.2022.891546)
Supplement: Supplementary file 2 [file Table_2.DOCX]

**Table S2.** Nutritional content and ingredients of the ultra-processed foods presented in the experiment

| **Ultra-processed foods** | | | | | | | | | |
| --- | --- | --- | --- | --- | --- | --- | --- | --- | --- |
| **Food** | **Ingredients** | **Energy (kJ)*** | **Sugar (g)***† | **Total fat (g)*** | **Saturated fat**  **(g)*** | **Trans fat (g)*** | **Sodium**  **(mg)*** | **Fiber (g)*** | **FSA**  **score** |
| ready-to-eat lasagna | Bolognese sauce (water, beef, tomato paste, modified starch, onion, smoked pork belly, margarine, sugar, salt, baked ham, garlic, potassium chloride, basil, chicken, basil, salsa, bay leaves, black pepper, rice flour, coconut oil, palm oil, milk fat, flavorings, natural flavorings, and flavor enhancers disodiumguanylate and disodiuminosinate), white sauce (water, milk cream, integral milk powder, margarine, modified starch, onion, smoked pork belly, salt, garlic, sugar, potassium chloride, rice flour, milk fat, oil coconut, palm oil, flavorings, natural aromas, and flavor enhancers disodium guanylate and disodium inosinate), enriched wheat flour, cheese, water, baked ham, egg yolk, egg. contains gluten. | 1787.39 | 0.00 | 30.24 | 8.67 | 0.63 | 1424.07 | 4.94 | 18 |
| chocolate discs | Sugar, cocoa mass, powdered milk, vegetable fat, corn starch, glucose syrup, cocoa butter, dextrin, salt, emulsifiers (soy lecithin and polyglycerol polyricinoleate), stabilizer gum arabic, inorganic coloring titanium dioxide, artificial colorings yellow, tartrazine, red 40, brilliant blue, natural coloring curcuma, flavorings, and glaze carnauba wax. | 2008.32 | 74.50§ | 20.00 | 18.00 | 0.00 | 65.00 | 0.00 | 25 |
| panettone with milk jam cream | Enriched wheat flour, milk jam flavor filling [corn glucose, sugar, water, milk jam, starch, salt, glycerin stabilizer, coloring: caramel iv (e150d/ins 150d), titanium dioxide (e171/ins 171) and urucum (e160b/ins 160b), potassium sorbate conservative, phosphoric acidulant, thickener carrageenan and flavoring], vegetable fat, sugar, whole egg and egg yolk, butter, glucose syrup, whole milk powder, malt extract, salt, emulsifiers: mono and diglycerides of fatty acids and soy lecithin, preservatives: calcium propionate and sorbic acid, flavoring and glycerin humectants. | 1359.75 | 12.00 | 12.50 | 7.13 | 0.00 | 95.00 | 1.13 | 13 |
| Brazilian cheese bread | Water, starch, cassava starch, pasteurized whole liquid egg, soybean oil, whey powder, cheese, salt, margarine, and flavoring similar to butter. | 1245.64 | 0.00 | 11.80 | 4.80 | 0.80 | 626.00 | 0.00 | 13 |
| ready-to-eat pizza | Dough (enriched wheat flour, water, soybean oil, dextrose, yeast, salt, malt extract, starch, glutathione, emulsifiers sodium stearoyl-2-lactyl lactate, polysorbate80), l-cysteine, cheese, tomato sauce, olive oil, and oregano. | 1025.98 | 0.00 | 11.30 | 4.41 | 0.00 | 805.19 | 2.34 | 12 |
| hamburger | Hamburger (beef, water, textured soy protein, hydrogenated vegetable fat, salt, condiment, maltodextrin, natural flavor, stabilizers sodium tripolyphosphate and sodium polyphosphate, flavor enhancer monosodium glutamate, antioxidant sodium erythorbate, and coloring beet red), bread (enriched wheat flour, water, sugar, sesame, hydrogenated vegetable fat, salt, emulsifiers mono and diglycerides of fatty acids, calcium stearoyl-2 lactated lactyl,polysorbate 80, and conservative calcium propionate) and cheddar-flavored processed cheese. | 1192.76 | 0.00 | 17.89 | 7.77 | 0.88 | 522.89 | 1.54 | 13 |
| ice cream | Water, sugar, vegetable fat, skimmed milk powder, inverted sugar, glucose syrup, whey, mono and diglycerides of fatty acids, stabilizers sodium alginate and disodium phosphate, flavorings, and colorings urucum and turmeric. | 681.34 | 25.00§ | 6.33 | 1.70 | 2.33 | 71.67 | 0.00 | 6 |
| chocolate bar | Sugar, cocoa butter, cocoa mass, milk powder, vegetable fat, cocoa powder, emulsifiers soy lecithin and polyglycerol polyricinoleate, and flavoring. | 2255.53 | 59.60§ | 30.30 | 17.50 | 0.00 | 77.10 | 2.20 | 23 |
| chocolate-covered marshmallow | Sugar, glucose syrup, vegetable fat, enriched wheat flour, cocoa powder, albumin, skimmed milk powder, agar-agar, soybean oil, whey powder and salt, humectant sorbitol, emulsifiers soy lecithin and polyglycerol polyricinoleate, chemical yeast baking soda, preservative potassium sorbate, and flavorings. | 1759.78 | 48.60 | 22.86 | 12.86 | 0.00 | 86.00 | 2.90 | 21 |
| margarine | Liquid and interesterified vegetable oils, water, salt, whey powder, skimmed milk powder, vitamin A, stabilizers mono and diglycerides, fatty acid and soy lecithin, preservative potassium sorbate and sodium benzoate, acidulant citric acid, antioxidants TBHQ, BHT, and EDTA, flavoring identical to natural flavors, and coloring beta-carotene, urucum and turmeric. | 2999.93 | 0.00 | 81.00 | 15.00 | 15.00 | 2.00 | 0.00 | 18 |
| bacon | Pork belly, water (9.98%), salt, sugar, stabilizer: sodium tripolyphosphate (INS 451i), antioxidant: sodium erythorbate (INS 316), natural smoke aroma and preservatives: sodium nitrite (INS 250) and sodium nitrate (INS 251). | 1546.60 | 0.00 | 34.00 | 11.00 | 0.00 | 1080.00 | 0.00 | 24 |
| microwave popcorn | Microwave popcorn corn, hydrogenated vegetable fat, refined salt, natural bacon flavoring, and monosodium glutamate flavor enhancer. | 1538.24 | 0.00 | 12.80 | 3.20 | 5.60 | 788.00 | 12.00 | 10 |
|  |  |  |  |  |  |  |  |  |  |
| cooked pork salami | Mechanically separated poultry meat, pork fat, pork meat, water, bovine meat, poultry meat, starch, soy protein, salt, sugar, garlic, cinnamon, coriander, nutmeg, acidity regulators sodium lactate and sodium citrate, stabilizers sodium pyrophosphate, sodium tripolyphosphate and sodium polyphosphate, flavor enhancer monosodium glutamate, antioxidant sodium isoascorbate, preservatives sodium nitrite and sodium nitrate, acidulant citric acid, coloring carmine, and flavorings. | 1143.27 | 0.00 | 19.00 | 5.39 | 0.00 | 1131.53 | 0.33 | 18 |
| cookie | Enriched wheat flour, wholegrain oatmeal, chocolate flavored drops (sugar, vegetable fat, whole milk powder, cocoa powder, whey powder, emulsifiers soy lecithin, sorbitantristearate and flavoring), sugar, vegetable fat, milk chocolate drops, whole milk powder, starch, salt, chemical yeast, ammonium bicarbonate, sodium bicarbonate and monocalcium phosphate, and flavoring and emulsifier soy lecithin. | 2048.20 | 37.50 | 22.50 | 12.50 | 0.00 | 145.00 | 0.00 | 25 |
| potato chips | Dehydrated potatoes, sunflower and corn vegetable oils, rice flour, wheat starch, maltodextrin, salt, emulsifiermono and diglyceride of fatty acids, and acidulant citric acid. | 2156.88 | 0.00 | 32.80 | 3.60 | 0.00 | 528.00 | 2.80 | 11 |
| gums | Sugar, glucose syrup, modified corn starch, flavorings, inorganic titanium dioxide dye, citric acid and fumaric acid acidulants, and artificial colors tartrazine yellow, crepuscular yellow, red 40 and brilliant blue. | 1554.96 | 92.00§ | 0.00 | 0.00 | 0.00 | 72.00 | 0.00 | 14 |
| nuggets | Chicken meat, mechanically recovered chicken meat, enriched wheat flour, water, chicken skin, rice flour, vegetable fat, corn derivatives, salt, soy protein, spices onion, garlic and white pepper, biological yeast, natural flavorings smoke, oregano, garlic, parsley, chicken and ginger, stabilizer guar gum, sodium polyphosphate, flavor enhancer monosodium glutamate, and antioxidant sodium isoascorbate. | 565.89 | 0.00 | 8.46 | 4.61 | 0.00 | 510.00 | 0.00 | 5 |
| popsicle | Water, sugar, glucose syrup, skim milk powder, vegetable fat, maltodextrin, whey, cocoa mass, vegetable oil, concentrated malt extract, cocoa, salt, stabilizers jatai gum, guar gum and carrageenan, acidity regulator baking soda, emulsifier mono and diglycerides of fatty acids, and flavoring. | 772.21 | 30.50§ | 5.76 | 2.88 | 0.00 | 54.24 | 0.00 | 9 |
| sausage | Pork, pork Fat, mechanically separated poultry meat, vegetable soy protein, water, salt, sugar, pepperoni, red pepper, collagen fiber, natural smoke and black pepper flavors, aromas identical to natural nutmeg, marjoram and bay leaf, flavor enhancer monosodium glutamate, antioxidants sodium erythorbate and ascorbic acid, stabilizersodium tripolyphosphate, acidulants lactic acid and citric, preservatives sodium nitrite and sodium nitrate, and natural coloring carmine. | 1354.32 | 0.00 | 28.00 | 9.20 | 0.00 | 1000.00 | 0.00 | 23 |
| cookie stuffed with vanilla | Wheat flour, sugar, vegetable fats (palm and palm kernel), 4.6% lean cocoa powder, wheat starch, corn syrup, leaveners (potassium bicarbonate, ammonium bicarbonate, and sodium bicarbonate), salt, emulsifiers(soy lecithin and sunflower lecithin), and flavorings (vanillin). | 1975.77 | 41.67 | 19.70 | 6.11 | 0.00 | 352.78 | 2.50 | 20 |
| starch chips | Cassava sprinkles, eggs, hydrogenated vegetable fat, refined salt, powdered whey, and flavoring. | 1769.39 | 0.00 | 16.67 | 2.67 | 4.67 | 933.33 | 5.67 | 12 |
| corn chips | Enriched wheat flour, mixed palm and soy oil, onion condiment (maltodextrin, salt, spices, sugar, flavor enhancers monosodium glutamate, disodium inosinate and disodium guanylate, gelling agent potassium chloride, antihumectant silicon dioxide and tricalcium phosphate, flavorings, acidulants citric acid and tartaric acid, and acidity regulator potassium citrate). | 2056.56 | 0.00 | 24.00 | 12.00 | 0.00 | 12.64 | 4.00 | 21 |
| hot dog | Mechanically separated chicken meat, mechanically recovered chicken meat, chicken meat, water, pork fat, pork meat, soy protein, pork skin, cassava starch, salt, dextrose, paprika, sugar, potassium chloride, natural spices, natural and identical to natural aromas, flavor enhancer monosodium glutamate, stabilizers disodium pyrophosphate and sodium tripolyphosphate, thickeners xanthan gum and carrageenan, antioxidant sodium erythorbate, preservatives sodium nitrite, sodium nitrate, and colorings carmine and urucum. | 1131.60 | 0.00 | 9.22 | 3.10 | 0.00 | 663.95 | 1.83 | 11 |
| wafer cookies | Sugar, interesterified vegetable fat, maltodextrin, enriched wheat flour, corn starch, salt, baking powder, emulsifier sodium lecithin, citric acid acidulant, natural coloring cochineal carmine, and flavoring. | 2050.16 | 30.66 | 22.66 | 10.66 | 0.00 | 86.66 | 3.66 | 17 |
| jelly | Sugar, gelatin, salt, vitamin C (sodium ascorbate), acidity regulators sodium citrate and fumaric acid, flavorings, edulcorants aspartame, sodium cyclamate, acesulfame k and sodium saccharin, andcoloringsordeaux S, sunset yellow, brilliant blue | 1421.20 | 58.00§ | 0.00 | 0.00 | 0.00 | 1880.00 | 0.00 | 24 |
| ready-to-drink chocolate milk | Reconstituted whey, reconstituted whole milk, cocoa syrup (water and cocoa), sugar, mixed soy and palm vegetable oil, barley malt extract, tricalcium phosphate, vitamins, salt, vitamin D3, stabilizers sodium carboxymethylcellulose, sodium citrate and carrageenan, emulsifiers mono and diglycerides of fatty acids and soy lecithin, and flavorings. | 349.03 | 12.00 | 2.55 | 1.20 | 0.00 | 80.00 | 0.00 | 3 |
| coke soda | Carbonated water, sugar, kola nut extract, caffeine, coloring caramel IV, acidulant phosphoric acid, and natural aroma. | 177.82 | 10.50§ | 0.00 | 0.00 | 0.00 | 5.00 | 0.00 | 2 |
| tortilla chips | Corn, vegetable oil and cheese-flavored prepared condiment (maltodextrin, salt, buttermilk, wheat flour, cheese, sugar, soy oil and cotton oil, spices, flavor enhancers monosodium glutamate, disodium inosinate and disodium guanylate, flavorings, acidulants disodium phosphate, citric acid and lactic acid, and artificial coloring sunset yellow and caramel). | 2006.40 | 0.00 | 25.20 | 10.40 | 0.00 | 492.00 | 0.00 | 20 |
| cookie stuffed with chocolate | Enriched wheat flour, sugar, hydrogenated vegetable fat, inverted sugar, calcium carbonate, cocoa, salt, colorings caramel III, carmine and synthetic beta-carotene, chemical yeast sodium bicarbonate, monocalcium phosphate and ammonium bicarbonate, flavorings, lecithin emulsifiers of soy and esters of diacetyl tartaric acid and mono and diglycerides of fatty acids and acidulant citric acid. | 2034.28 | 36.67 | 21.66 | 9.00 | 1.00 | 216.67 | 4.00 | 19 |
| breakfast cereals | Corn flour blend (whole grain yellow corn flour, degerminated yellow corn flour), sugar, wheat flour, whole grain oat flour, contains 2% or less of oat fiber, hydrogenated vegetable oil (coconut, soybean and/or cottonseed), salt, soluble corn fiber, natural flavor, red 40, turmeric extract color, blue 1, yellow 6, annatto extract color, BHTfor freshness,vitamins and minerals: vitamin c (sodium ascorbate and ascorbic acid), niacinamide, reduced iron, zinc oxide, vitamin B6 (pyridoxine hydrochloride), vitamin B2 (riboflavin), vitamin B1 (thiamin hydrochloride), vitamin A palmitate, folic acid, vitamin B12, and vitamin D3. | 1588.40 | 40.00 | 2.67 | 1.33 | 0.00 | 410.00 | 3.00 | 13 |
| instant noodles | Enriched wheat flour, vegetable fat, salt, rice flour, manioc starch, dairy compound, chicken flakes, chicken-flavored prepared flavor based on yeast extract, tomato flakes, corn flakes, parsley flakes, garlic-flavored seasoning, cheese-flavored seasoning, chicken-flavored seasoning, coriander powder, turmeric powder, garlic powder, pepper seasoning, celery powder, flavor enhancers monosodium glutamate, disodium inosinate,and disodium guanylate, thickener xanthan gum and guar gum, acidity regulators potassium carbonate and sodium carbonate, flavoring, coloring beta-carotene and titanium dioxide, stabilizer sodium tripolyphosphate, acidulant citric acid, and antihumectant silicon dioxide. | 1822.94 | 0.00 | 19.44 | 8.89 | 0.00 | 1684.70 | 3.60 | 18 |
| grape soda | Carbonated water, sugar, grape and lemon juices, synthetic aroma identical to natural, acidity regulators citric acid and sodium citrate, preservatives sodium benzoate and potassium sorbate, and artificial colorings amaranth, brilliant blue,and tartrazine. | 204.82 | 12.00§ | 0.00 | 0.00 | 0.00 | 12.00 | 0.00 | 2 |

FSA, Food Standards Agency.

*Per 100 g or 100 mL.

† Considered free sugar values.

§ Considered the total carbohydrate value because the amount of free sugars was unavailable.
