# Supplementary material for: Ultra-Processed Foods Elicit Higher Approach Motivation Than Unprocessed and Minimally Processed Foods
Source: Front Public Health. 2022 Jun 21;10:891546. doi: 10.3389/fpubh.2022.891546 (PMC9253546; doi:10.3389/fpubh.2022.891546)
Supplement: Supplementary file 4 [file Table_4.DOCX]

**Table S4.** Intention to consume ratings for each participant

| **Intention to consume ratings for each participant** | | | | | |
| --- | --- | --- | --- | --- | --- |
|  | **Unprocessed/minimally processed foods** | | **Ultraprocessed foods** | |  |
| **Participant** | **Mean** | **SD** | **Mean** | **SD** | **Δ** |
| **Participant 1** | **5.13** | **3.48** | **6.00** | **3.42** | **0.88** |
| **Participant 2** | **6.88** | **0.99** | **4.50** | **3.16** | **-2.38** |
| **Participant 3** | **5.63** | **2.77** | **6.50** | **2.73** | **0.88** |
| **Participant 4** | **3.25** | **3.41** | **6.25** | **2.92** | **3.00** |
| **Participant 5** | **0.25** | **0.46** | **0.38** | **0.52** | **0.13** |
| **Participant 6** | **6.88** | **1.64** | **6.00** | **2.88** | **-0.88** |
| **Participant 7** | **4.25** | **3.73** | **3.38** | **3.66** | **-0.88** |
| **Participant 8** | **0.75** | **1.16** | **1.75** | **1.75** | **1.00** |
| **Participant 9** | **4.25** | **3.28** | **4.50** | **3.85** | **0.25** |
| **Participant 10** | **3.13** | **3.68** | **2.88** | **3.18** | **-0.25** |
| **Participant 11** | **5.00** | **3.34** | **5.25** | **3.65** | **0.25** |
| **Participant 12** | **3.75** | **3.33** | **5.63** | **3.11** | **1.88** |
| **Participant 13** | **5.00** | **2.67** | **5.38** | **2.92** | **0.38** |
| **Participant 14** | **3.25** | **2.96** | **4.00** | **2.51** | **0.75** |
| **Participant 15** | **5.00** | **1.85** | **5.00** | **1.93** | **0.00** |
| **Participant 16** | **4.75** | **2.31** | **6.25** | **1.39** | **1.50** |
| **Participant 17** | **6.13** | **0.99** | **3.50** | **1.85** | **-2.63** |
| **Participant 18** | **3.88** | **2.70** | **3.50** | **2.45** | **-0.38** |
| **Participant 19** | **7.88** | **0.35** | **3.00** | **2.83** | **-4.88** |
| **Participant 20** | **4.00** | **2.67** | **5.00** | **3.02** | **1.00** |
| **Participant 21** | **5.88** | **2.70** | **6.25** | **2.60** | **0.38** |
| **Participant 22** | **3.50** | **3.02** | **3.38** | **3.38** | **-0.13** |
| **Participant 23** | **3.88** | **3.14** | **5.75** | **3.28** | **1.88** |
| **Participant 24** | **2.88** | **2.03** | **3.13** | **2.42** | **0.25** |
| **Participant 25** | **3.13** | **2.30** | **2.50** | **2.83** | **-0.63** |
| **Participant 26** | **3.00** | **2.00** | **4.63** | **3.20** | **1.63** |
| **Participant 27** | **5.38** | **2.97** | **7.88** | **0.35** | **2.50** |
| **Participant 28** | **3.75** | **3.62** | **6.13** | **2.03** | **2.38** |
| **Participant 29** | **3.38** | **1.85** | **4.13** | **3.31** | **0.75** |
| **Participant 30** | **5.25** | **1.58** | **6.25** | **1.16** | **1.00** |
| **Participant 31** | **3.00** | **1.60** | **1.75** | **2.19** | **-1.25** |
| **Participant 32** | **0.63** | **0.52** | **1.00** | **0.53** | **0.38** |
| **Participant 33** | **2.50** | **2.45** | **5.75** | **2.55** | **3.25** |
| **Participant 34** | **3.88** | **1.96** | **5.00** | **2.00** | **1.13** |
| **Participant 35** | **4.13** | **1.96** | **5.25** | **3.41** | **1.13** |
| **Participant 36** | **3.25** | **2.96** | **5.88** | **2.90** | **2.63** |
| **Participant 37** | **3.13** | **3.00** | **6.00** | **2.51** | **2.88** |
| **Participant 38** | **6.75** | **1.04** | **6.50** | **1.51** | **-0.25** |
| **Participant 39** | **7.13** | **0.83** | **6.63** | **2.77** | **-0.50** |
| **Participant 40** | **1.38** | **2.88** | **2.38** | **2.20** | **1.00** |
| **Participant 41** | **3.75** | **2.31** | **4.00** | **2.93** | **0.25** |
| **Participant 42** | **5.13** | **1.96** | **5.38** | **1.77** | **0.25** |
| **Participant 43** | **4.25** | **3.65** | **4.50** | **3.46** | **0.25** |
| **Participant 44** | **2.00** | **2.78** | **4.25** | **3.33** | **2.25** |
| **Participant 45** | **1.13** | **2.10** | **4.75** | **2.76** | **3.63** |
| **Participant 46** | **1.25** | **1.83** | **2.50** | **2.27** | **1.25** |
| **Participant 47** | **5.88** | **3.04** | **7.00** | **1.07** | **1.13** |
| **Participant 48** | **6.88** | **1.13** | **6.38** | **0.92** | **-0.50** |
| **Participant 49** | **3.88** | **2.85** | **3.88** | **3.27** | **0.00** |
| **Participant 50** | **6.63** | **2.39** | **6.75** | **2.82** | **0.13** |
| **Participant 51** | **1.38** | **2.00** | **4.00** | **2.73** | **2.63** |
| **Participant 52** | **2.88** | **1.81** | **4.75** | **2.60** | **1.88** |
| **Participant 53** | **0.63** | **0.74** | **1.38** | **1.60** | **0.75** |
| **Participant 54** | **0.63** | **0.74** | **0.38** | **0.74** | **-0.25** |
| **Participant 55** | **2.38** | **2.92** | **3.50** | **3.34** | **1.13** |
| **Participant 56** | **4.25** | **2.71** | **3.88** | **2.70** | **-0.38** |
| **Participant 57** | **4.38** | **2.83** | **6.38** | **2.67** | **2.00** |
| **Participant 58** | **5.13** | **3.14** | **6.25** | **3.28** | **1.13** |
| **Participant 59** | **6.50** | **1.31** | **3.38** | **2.45** | **-3.13** |
| **Participant 60** | **5.38** | **2.77** | **4.75** | **3.01** | **-0.63** |
| **Participant 61** | **4.63** | **3.42** | **6.25** | **2.76** | **1.63** |
| **Participant 62** | **3.38** | **3.02** | **4.88** | **1.96** | **1.50** |
| **Participant 63** | **6.00** | **1.69** | **7.25** | **0.89** | **1.25** |
| **Participant 64** | **5.25** | **3.65** | **4.63** | **3.29** | **-0.63** |
| **Participant 65** | **0.63** | **1.41** | **5.63** | **2.62** | **5.00** |
| **Participant 66** | **3.00** | **3.38** | **1.13** | **1.73** | **-1.88** |
| **Participant 67** | **1.50** | **2.14** | **5.13** | **2.90** | **3.63** |
| **Participant 68** | **4.88** | **1.89** | **4.75** | **2.19** | **-0.13** |
| **Participant 69** | **1.00** | **2.14** | **5.13** | **3.27** | **4.13** |
| **Participant 70** | **4.13** | **3.64** | **6.75** | **1.83** | **2.63** |
| **Participant 71** | **1.38** | **1.06** | **2.13** | **0.99** | **0.75** |
| **Participant 72** | **4.13** | **2.36** | **3.38** | **2.00** | **-0.75** |
| **Participant 73** | **2.88** | **3.14** | **2.88** | **2.36** | **0.00** |
| **Participant 74** | **3.25** | **3.06** | **4.50** | **3.85** | **1.25** |
| **Participant 75** | **6.00** | **2.20** | **5.88** | **2.17** | **-0.13** |
| **Participant 76** | **4.75** | **3.37** | **5.50** | **2.78** | **0.75** |
| **Participant 77** | **4.13** | **2.10** | **4.63** | **2.07** | **0.50** |
| **Participant 78** | **2.00** | **2.62** | **2.38** | **1.60** | **0.38** |
| **Participant 79** | **3.25** | **3.11** | **6.13** | **3.00** | **2.88** |
| **Participant 80** | **2.63** | **3.58** | **3.00** | **2.67** | **0.38** |
| **Participant 81** | **0.38** | **0.74** | **0.13** | **0.35** | **-0.25** |
| **Participant 82** | **6.50** | **2.83** | **5.63** | **3.34** | **-0.88** |
| **Participant 83** | **5.25** | **3.06** | **5.00** | **3.07** | **-0.25** |
| **Participant 84** | **5.38** | **3.34** | **1.38** | **2.77** | **-4.00** |
| **Participant 85** | **3.13** | **2.75** | **5.50** | **2.51** | **2.38** |
| **Participant 86** | **4.75** | **1.67** | **4.38** | **2.20** | **-0.38** |
| **Participant 87** | **3.38** | **3.81** | **6.75** | **2.76** | **3.38** |
| **Participant 88** | **3.00** | **2.88** | **2.38** | **2.50** | **-0.63** |
| **Participant 89** | **2.88** | **2.23** | **4.25** | **3.11** | **1.38** |
| **Participant 90** | **4.75** | **2.25** | **6.63** | **2.88** | **1.88** |
| **Participant 91** | **0.13** | **0.35** | **0.63** | **1.77** | **0.50** |
| **Participant 92** | **0.38** | **0.74** | **2.63** | **2.72** | **2.25** |
| **Participant 93** | **5.63** | **2.33** | **5.00** | **3.42** | **-0.63** |
| **Participant 94** | **4.00** | **2.73** | **4.13** | **2.75** | **0.13** |
| **Participant 95** | **5.00** | **2.78** | **5.25** | **2.55** | **0.25** |
| **Participant 96** | **4.38** | **1.30** | **3.38** | **2.33** | **-1.00** |
| **Participant 97** | **5.75** | **2.92** | **5.25** | **3.28** | **-0.50** |
| **Participant 98** | **2.13** | **0.64** | **1.13** | **1.25** | **-1.00** |
| **Participant 99** | **3.00** | **3.16** | **2.38** | **2.50** | **-0.63** |
| **Participant 100** | **2.88** | **3.14** | **5.00** | **2.98** | **2.13** |
| **Participant 101** | **3.25** | **1.83** | **6.13** | **2.85** | **2.88** |
| **Participant 102** | **4.50** | **2.62** | **5.88** | **1.81** | **1.38** |
| **Participant 103** | **2.88** | **2.53** | **2.38** | **2.77** | **-0.50** |
| **Participant 104** | **3.50** | **0.76** | **4.13** | **2.23** | **0.63** |
| **Participant 105** | **5.25** | **2.55** | **3.88** | **3.60** | **-1.38** |
| **Participant 106** | **2.00** | **1.41** | **4.00** | **2.73** | **2.00** |
| **Participant 107** | **4.50** | **3.16** | **4.25** | **3.01** | **-0.25** |
| **Participant 108** | **6.00** | **3.42** | **2.88** | **3.56** | **-3.13** |
| **Participant 109** | **3.25** | **2.55** | **5.00** | **1.85** | **1.75** |
| **Participant 110** | **2.50** | **2.73** | **5.00** | **3.16** | **2.50** |
| **Participant 111** | **5.00** | **3.66** | **6.75** | **1.28** | **1.75** |
| **Participant 112** | **6.38** | **1.06** | **6.75** | **1.16** | **0.38** |
| **Participant 113** | **5.13** | **3.36** | **7.63** | **0.74** | **2.50** |
| **Participant 114** | **5.13** | **2.47** | **6.75** | **2.05** | **1.63** |
| **Participant 115** | **0.50** | **1.07** | **2.25** | **3.11** | **1.75** |
| **Participant 116** | **7.38** | **0.74** | **7.25** | **1.04** | **-0.13** |
| **Participant 117** | **5.13** | **3.27** | **6.25** | **2.76** | **1.13** |
| **Participant 118** | **2.88** | **2.85** | **7.50** | **0.76** | **4.63** |
| **Participant 119** | **6.25** | **2.71** | **2.00** | **3.70** | **-4.25** |
| **Participant 120** | **3.88** | **2.23** | **4.25** | **2.25** | **0.38** |
| **Participant 121** | **4.38** | **3.85** | **5.13** | **3.98** | **0.75** |
| **Participant 122** | **4.13** | **2.95** | **3.38** | **3.96** | **-0.75** |
| **Participant 123** | **3.25** | **1.28** | **4.88** | **1.81** | **1.63** |
| **Participant 124** | **3.13** | **2.70** | **5.00** | **2.83** | **1.88** |
| **Participant 125** | **1.13** | **2.42** | **6.63** | **1.85** | **5.50** |
| **Participant 126** | **5.38** | **1.06** | **3.88** | **2.64** | **-1.50** |
| **Participant 127** | **7.50** | **0.53** | **5.38** | **2.77** | **-2.13** |
| **Participant 128** | **2.50** | **3.46** | **3.13** | **4.05** | **0.63** |
| **Participant 129** | **5.88** | **2.90** | **6.00** | **2.83** | **0.13** |
| **Participant 130** | **5.13** | **1.96** | **6.00** | **2.45** | **0.88** |
| **Participant 131** | **4.13** | **2.59** | **3.50** | **2.62** | **-0.63** |
| **Participant 132** | **7.50** | **1.07** | **0.63** | **1.41** | **-6.88** |
| **Participant 133** | **7.50** | **0.53** | **3.25** | **3.06** | **-4.25** |
| **Participant 134** | **2.75** | **3.28** | **2.13** | **3.36** | **-0.63** |
| **Participant 135** | **5.00** | **3.38** | **3.75** | **3.49** | **-1.25** |
| **Participant 136** | **1.50** | **2.33** | **4.13** | **2.95** | **2.63** |
| **Participant 137** | **5.13** | **2.36** | **1.75** | **2.49** | **-3.38** |
| **Participant 138** | **3.25** | **2.66** | **4.63** | **2.20** | **1.38** |
| **Participant 139** | **4.25** | **2.82** | **4.75** | **2.87** | **0.50** |
| **Participant 140** | **5.13** | **2.30** | **5.13** | **1.96** | **0.00** |
| **Participant 141** | **6.00** | **2.27** | **3.88** | **3.72** | **-2.13** |
| **Participant 142** | **3.88** | **3.04** | **3.00** | **3.16** | **-0.88** |
| **Participant 143** | **6.13** | **2.23** | **6.00** | **1.85** | **-0.13** |
| **Participant 144** | **3.13** | **3.36** | **4.75** | **3.28** | **1.63** |
| **Participant 145** | **2.50** | **3.02** | **3.50** | **2.20** | **1.00** |
| **Participant 146** | **5.75** | **2.19** | **6.75** | **2.82** | **1.00** |
| **Participant 147** | **0.38** | **0.52** | **1.25** | **0.89** | **0.88** |
| **Participant 148** | **1.50** | **1.20** | **4.00** | **3.07** | **2.50** |
| **Participant 149** | **2.88** | **3.48** | **4.50** | **3.38** | **1.63** |
| **Participant 150** | **3.75** | **2.82** | **4.75** | **2.96** | **1.00** |
| **Participant 151** | **5.00** | **3.02** | **4.13** | **2.59** | **-0.88** |
| **Participant 152** | **3.00** | **2.51** | **6.75** | **1.58** | **3.75** |
| **Participant 153** | **3.25** | **1.16** | **3.50** | **0.93** | **0.25** |
| **Participant 154** | **0.75** | **0.89** | **6.50** | **2.45** | **5.75** |
| **Participant 155** | **3.63** | **3.58** | **6.38** | **1.69** | **2.75** |
| **Participant 156** | **3.25** | **2.60** | **4.63** | **1.30** | **1.38** |
| **Participant 157** | **1.00** | **1.77** | **3.13** | **2.03** | **2.13** |
| **Participant 158** | **3.50** | **2.83** | **5.38** | **2.92** | **1.88** |
| **Participant 159** | **3.63** | **2.33** | **4.00** | **1.93** | **0.38** |
| **Participant 160** | **6.88** | **1.55** | **4.75** | **3.15** | **-2.13** |
| **Participant 161** | **2.00** | **2.33** | **1.75** | **2.76** | **-0.25** |
| **Participant 162** | **2.75** | **2.96** | **5.13** | **3.14** | **2.38** |
| **Participant 163** | **2.88** | **2.03** | **3.75** | **2.49** | **0.88** |
| **Participant 164** | **0.50** | **0.76** | **6.00** | **3.16** | **5.50** |
| **Participant 165** | **1.25** | **1.39** | **4.38** | **2.20** | **3.13** |
| **Participant 166** | **3.88** | **3.18** | **6.00** | **2.14** | **2.13** |
| **Participant 167** | **3.13** | **3.31** | **6.88** | **2.23** | **3.75** |
| **Participant 168** | **0.75** | **1.49** | **5.13** | **3.56** | **4.38** |
| **Participant 169** | **3.13** | **2.42** | **7.00** | **1.20** | **3.88** |
| **Participant 170** | **2.88** | **2.75** | **6.25** | **1.83** | **3.38** |
| **Participant 171** | **2.63** | **2.72** | **7.25** | **1.16** | **4.63** |
| **Participant 172** | **4.88** | **4.05** | **7.75** | **0.46** | **2.88** |
| **Participant 173** | **6.75** | **2.43** | **4.88** | **2.75** | **-1.88** |
| **Participant 174** | **0.13** | **0.35** | **4.50** | **2.98** | **4.38** |
